# Supplementary material for: Workplace Assessment Scale: Pilot Validation Study
Source: Int J Environ Res Public Health. 2022 Sep 29;19(19):12408. doi: 10.3390/ijerph191912408 (PMC9566622; doi:10.3390/ijerph191912408)
Supplement: Supplementary file 1 [file ijerph-19-12408-s001.zip › File S1 - Case Vignettes_FINAL.pdf]

Case Vignette 1 – Jane

The hardest part of sharing your story of trauma is simply the beginning. How and where do you possibly start the conversation of your loss of self, loss of value, loss of security, loss of happiness? I have been a first responder passionately serving several communities for over 21 years. For me the "iceberg" moment was one year ago when I took up a new position. I had safety concerns about what I was being asked to do but management did not support my concerns. My supervisor turned up unannounced at work and was relentlessly antagonizing, yelling at me, and berating me in front of my team and the public. One of the other first responders present jumped up and ran to put a table between herself and the supervisor. In her statement she said she was frightened for her safety and "didn't know what was going to happen." I received an email from my Deputy Chief condemning me for my "unprofessional behaviour" and warned me to not behave like that again. I also received a voice message from my supervisor stating that I had to attend a mandatory disciplinary meeting where I would be "escorted to the meeting". I was emotionally devastated so I went off on sick leave.

My employer informed me that they would not start an investigation until I returned to work. During my attempt to return to work my employer refused to give me any modified duties. I was told I had to return to full duties or not return at all. They made the return-to-work process hostile and difficult. On the day I returned to work to complete a training day they stated they had nothing prepared for me. They refused to let me go get a coffee and instead made me sit in the office for six hours with no work.

My first shift back to work my employer sent me an email with an "Exit Package" asking me to consider leaving the organization. This sent me into a deep depression, and I lost myself completely. I struggled to even get out of bed. So, once again I was forced to go off on sick leave. I again worked very hard to heal and become strong enough to handle returning to my workplace. Once again, my first shift back I was sent an email stating I had to attend a performance review for disciplinary action. I felt immediately vulnerable and felt attacked. The management team put a written disciplinary letter on my file stating that my Chief was told by "someone" that "they heard me say something that could have been perceived as derogatory towards the organization.". They refused to say who, what or when. They also claimed that in the original correspondence I stated that my Chief wasn't being "clever". The third thing was that during my initial return to work training I had "maliciously and fraudulently" lied about completing online training. On the day that I did my return-to-work they questioned me about the online training that I stated I completed. As soon as I was notified that it was not showing up despite my belief that I had completed it, I went online and completed what they wanted. It was still completed 45 days before the due date. However, management did not accept this and stated I had intentionally lied about doing it and stated my intent was "malicious". I couldn't believe they would question my integrity and professionalism. When I explained the confusion that stress and PTSD can cause they responded with "if there are no restrictions on your file then as far as we are concerned your PTSD is cured". This from a first responder organization.

Now, here I am off work again feeling completely defeated, worthless, and abandoned. I have lost all the confidence I once had. I feel completely unsafe in my workplace and worry constantly that they will find a way to fire me. How can first responders possibly continue to fight the daily battles out on the frontlines when there is this lethal battle going on behind the scenes?

For people like me there is little hope for a safe or supportive future, and this can lead to unspeakable desperation. I'm lost and exhausted. I feel like a fool for being so dedicated and for sacrificing so much thinking it was valued and appreciated. So stupid for thinking that if one day I needed help my employer would be there to support me. I just hope that sharing my suffering will help change the system and force these heartless bullies to change because they will never change on just moral grounds.

Case Vignette 2 - Mark

My story started in April 2001 which was when I started my career as a first responder. I was excited to be part of something that I was missing in my life, a family. The service became my family, and it would consume my life. The brotherhood and sisterhood that I was part of became the most important part of my life.

Over the course of 20 years, I worked alongside my colleagues witnessing some of the worst calls I could've imagined. Sadly, I've witnessed carnage from a traffic accident, victims of sexual assaults, children being human trafficked, murder victims, and several suicides. With all the calls that I witnessed, the ones that would have a debilitating impact on me were the suicides.

I didn't know it at the time, but the accumulation of these calls would later lead to my Post Traumatic Stress Injury. I became very depressed after 20 years of being on the job, and I was ashamed to admit that I had a problem. PTSD impacted my sleep, I became very angry around people, I started drinking alcohol heavily and I started having a fascination with committing suicide. I felt that this was my only way out of feeling the way that I was. I became afraid of responding to calls because I did not want to see more carnage, and I didn't want to live anymore.

After years of feeling this way, I went off of work. Shortly after, I started seeing a psychologist weekly, and a psychiatrist every other week. I was prescribed anti-depressants, and anxiety medication. I was given the diagnosis of having PTSD. These treatments would give me greater stability in my life. I was happy to get the help that I needed, and I was making progress, but what happened next, I couldn't have prepared for.

My employer started coming after me with policy violations. They accused me invalid notebook entries such as underlining entries improperly, using a force vehicle for personal use, obtaining unsanctioned car washes and improper time scheduling. As a result of these accusations, the employer wants to dismiss me from work. This is the penalty they are

seeking for the policy violations. So, as I explained before, when I started being a first responder, the service became my family. After the accusations started, I felt my family was abandoning me and didn't want me in their lives. I felt targeted, betrayed, and hated. My colleagues turned their backs on me. People I was close to wouldn't even acknowledge me in public.

So, with all the things my service was doing to me, I became more suicidal than ever. I was fortunate enough to be surrounded by the professionals I have. They were able to deescalate the situation with me and they got me back on track. I’ve been in therapy dealing with my PTSI for a year and a half now. I’ve learned a lot about myself and my injury. I’m no longer ashamed of admitting that I have an issue. I’ve accepted the fact that I can’t be a first responder anymore, and I’m good with this decision.

But my issues now are that my service clearly has no use for me, and they are targeting me to either fire me or force me to quit. Sadly, their actions have a tremendous negative impact on my rehab.
