# Supplementary material for: Workplace Assessment Scale: Pilot Validation Study
Source: Int J Environ Res Public Health. 2022 Sep 29;19(19):12408. doi: 10.3390/ijerph191912408 (PMC9566622; doi:10.3390/ijerph191912408)
Supplement: Supplementary file 1 [file ijerph-19-12408-s001.zip › Table S1. Study outcome measures and timing of administration_FINAL.pdf]

Table S1. Study outcome measures and timing of administration

Measures	Indication	Administration	Timing			
			Baseline	Week 4	Week 8	Week 12
Montgomery-Asberg Depression Rating Scale (MADRS)	Depression	Interview	X	-	-	-
Clinician-Administered PTSD Scale for DSM-5 (CAPS-5)	PTSD	Interview	X	-	-	-
World Health Organization – Alcohol, Smoking and Substance Involvement Screening Test (WHO – ASSIST)	Substance Use	Interview	X	-	-	-
Maslach Burnout Inventory – Human Services Survey (MBI-HSS)	Burnout	Self-Report	X	-	-	-
Adverse Childhood Experiences Scale (ACE-Q)	Childhood Maltreatment	Self-Report	X	-	-	-
Workplace assessment scale (WAS)	Workplace Satisfaction	Self-Report	X	-	-	-
Oslo Social Support Scale (OSSS-3)	Social Support	Self-Report	X	-	-	-
Patient Health Questionnaire – 9 (PHQ-9)	Depression	Self-Report	X	X	X	X
PTSD Checklist for DSM-5 (PCL-5)	PTSD	Self-Report	X	X	X	X
Alcohol Use Disorders	Alcohol Use	Self-Report	X	X	X	X

Identification Test (AUDIT)						
Drug Abuse Screening Test (DAST-10)	Substance Use	Self-Report	X	X	X	X
World Health Organization Quality of Life (WHOQOL-BREF)	Quality of Life	Self-Report	X	X	X	X
Generalized Anxiety Disorder-7 (GAD-7)	Anxiety	Self-Report	X	X	X	X
Warwick Edinburgh Mental Wellbeing Scale (WEMWBS)	Wellbeing	Self-Report	X	X	X	X
Workplace Productivity and Impairment (WPAI)	Workplace Productivity	Self-Report	X	X	X	X
Columbia-Suicide Severity Rating Scale (C-SSRS)	Suicidality	Self-Report	X	X	X	X
